# Journey of Hope for Patients with Fibromyalgia: From Diagnosis to Self-Management—A Qualitative Study

**DOI:** 10.3390/healthcare13020142

**Published:** 2025-01-13

**Authors:** Amal Aldarwesh

**Affiliations:** Optometry Department, College of Applied Medical Sciences, King Saud University, Riyadh 11433, Saudi Arabia; aaldarweesh@ksu.edu.sa

**Keywords:** fibromyalgia, syndrome, qualitative, content analysis, self-management, coping

## Abstract

Background/Objectives: Fibromyalgia syndrome (FMS) is a chronic, debilitating condition characterized by widespread pain, fatigue, and psychological distress. There is a lack of qualitative studies on the unique experiences of patients with FMS in Arab countries, particularly through social media. Despite the availability of diagnostic criteria, diagnosing and managing patients remains challenging. This study aimed to describe the experiences of patients with FMS in Arab countries, their understanding of the illness, and perceptions of treatment. Methods: A qualitative study was conducted using a content analysis of patients’ narratives published in a supportive group, describing their experiences with fibromyalgia. The dataset included 2305 quotes from 192 main posts and 2113 comments collected between 2019 and 2024. Results: The analysis of the posts and associated comments revealed six main themes: patients’ experiences with the syndrome, symptoms, searching for a doctor, pharmacological management, self-management, and the impact of fibromyalgia and peer support. Most posts and comments focused on patients’ experiences with self-management approaches and coping strategies, highlighting significant noncompliance with therapeutic modalities. Factors influencing patients’ experiences and decisions included their relationship with physicians, medication side effects, personal fears, and physical and mental health. Conclusions: Patients with FMS in Arab countries face similar challenges to those in other regions, including physical, psychological, social, and economic impacts. Many patients reject conventional therapeutic management strategies and adopt coping mechanisms to mitigate adverse effects and healthcare costs. The findings suggest that the physician–patient relationship, as well as the physician’s knowledge and attitude toward fibromyalgia syndrome, are the cornerstones of gaining patients’ trust.

## 1. Introduction

Fibromyalgia syndrome (FMS) is a clinical condition characterized by a cycle of chronic pain, sleep disturbance, and fatigue that often leads to mood disorders [1]. The exact etiology remains unclear; however, research suggests that FMS may be due to abnormalities in pain processing within the brain [1,2]. This includes elevated levels of excitatory neurotransmitters, such as glutamate and substance P, leading to central nervous system (CNS) sensitization and increased pain [1,2]. Prevalence studies conducted in Asia, Europe, North America, and Turkey indicate a range of 1.62% to 9.3% within the general population [2,3]. More recent studies using varied methodologies reported a higher prevalence of FMS in the Middle East. In Saudi Arabia, a systematic review and meta-analysis study included 4967 patients, using several diagnostic criteria and instruments, which reported a notably higher prevalence of FMS, at 13.4%, with more women affected than men [4]. A similar prevalence rate of 13.2% was reported using a self-reported fibromyalgia rapid screening tool in a cross-sectional online survey completed by 691 Egyptian physicians with a female-to-male ratio of 3.8:1. In Jordan, a cross-sectional study conducted using the American College of Rheumatology (ACR) criteria and a survey of 956 participants found a 26.5% prevalence of FMS in the study population, with female gender associated with the syndrome. [4,5,6]. Diagnosis has traditionally been based on the ACR 1990 criteria, which require widespread pain for at least 3 months and tenderness at 11 of 18 specific tender points [7]. This guideline was later modified to simplify diagnosis by excluding tender points, instead incorporating the Widespread Pain Index and Symptom Severity Scale to assess patients. In addition, symptoms must have persisted for at least 3 months in the absence of any other disorder that would explain the pain [8]. Despite these established diagnostic criteria, the average time from symptom onset to diagnosis is approximately 6 years, during which patients typically consult multiple specialists [9,10]. This delayed diagnosis imposes a socioeconomic burden on both healthcare systems and patients, and is associated with poorer patient outcomes, including increased symptom severity, as well as deteriorated mental health and quality of life [11,12].

The U.S. Food and Drug Administration has approved three medications for FMS: the antiepileptic drug, pregabalin, and two antidepressants, which are duloxetine and milnacipran. However, other off-label antidepressants are prescribed for FMS, such as tricyclic antidepressants, amitriptyline, and selective serotonin reuptake inhibitors such as citalopram, and the muscle relaxant, tricyclic derivative cyclobenzaprine [13]. Non-pharmacological recommendations for FMS patients include exercise programs, cognitive behavior therapy, hyperbaric oxygen therapy, acupuncture, and lifestyle modifications including regular tailored exercise, a well-balanced diet, and engagement in stress-release activities such as breathing exercises and mindfulness [13,14].

Studies examining patients’ experiences with FMS—from diagnosis through to treatment and the associated physical and psychological suffering—have primarily employed quantitative approaches, often using tools such as questionnaires [9,15,16,17]. Qualitative studies on chronic conditions like FMS are valuable, as they provide an in-depth view of patients’ experiences and offer insights into their perspectives on the condition and its therapeutic management. In the Arab region, few qualitative studies have explored the challenges faced by patients with FMS in managing this debilitating syndrome. Social media platforms provide a small community-like setting where patients can share experiences, values, and beliefs and receive peer support. Numerous studies have conducted qualitative content analyses of chronic conditions, including breast cancer and diabetes mellitus, through Facebook [18,19,20,21]. This qualitative study was initiated to explore patients’ unique experiences and gain insights into their perspectives by analyzing content from a patient support group on Facebook. A more comprehensive understanding can help healthcare professionals address patient concerns during treatment and establish a foundation for effective communication and mutual understanding.

## 2. Methods

### 2.1. Study Design and Data Collection

This study is based on qualitative design, employing a mixed deductive–inductive content analysis. The data for this study were obtained from the public Facebook page of the Jordanian Society of Fibromyalgia Support Group. At the time of data collection, the membership on this page was 4610. The dataset consisted of 2305 quotes comprising 192 main posts and 2113 comments, collected chronologically for analysis from posts published between 2019 and 2024. The recent posts were retrieved until they reached saturation by posts published in 2019. Most of the posts and comments were in Arabic; however, English posts were also included in the analysis. This group mostly included women who self-reported being diagnosed with FMS and living in Arab countries. It is worth mentioning that the nature of this study does not allow for examining the demographic information or the diagnosis criteria of those patients.

### 2.2. Data Analysis

Qualitative data were analyzed using both inductive and deductive content analyses. The unit of analysis was each individual’s narrative post or comment on the Facebook page. The data were tabulated in Thematic Coder 1.0, an Excel sheet developed by Chan (2012), available from www.derwinchan.com [22]. Relevant quotes in the project data were coded at a theme node. A node represents a collection of quotes from the data (i.e., Facebook posts) on a specific theme.

For example, suppose the following quote is added from one post: “duloxetine makes me sleepy”. In that case, the whole sentence is equal to one node, and it is linked to the theme “pharmacological management of fibromyalgia”. If a post or comment was too long and contained several ideas, codes, or themes, the sentence was broken into multiple quotes to accurately match the theme, as in the following hypothetical example: “duloxetine makes me sleepy, and I am thinking of stopping the drug (node 1, pharmacological management theme). I am disappointed with my physician; he does not listen to me. Could you please recommend a rheumatologist with whom you have had a good experience? (nodes 2 &3, relationship with physician and searching for a doctor theme) For now, I started light exercises and a keto diet (node 4, Self-management theme).” In qualitative data analysis software like Nvivo, tree nodes organize all known information about a particular theme or code hierarchically [23]. The thematic coder Excel sheet used in this study calculates the percentage, number, and coverage of nodes, in addition to the number of words under each theme. The higher the number of nodes in a theme, the higher the coverage of that theme in the data. Quantitative data were also calculated on the number of posts that discuss symptoms, medications, types of exercises, and dietary modification. After the initial data collection of 500 quotes, the researcher thoroughly examined the data to familiarize herself with the patterns and generated an initial coding framework. Data were retrieved until saturation was achieved, when no new codes or themes were generated as participants continued to repetitively post similar thoughts, questions, and concerns. The author repeatedly read the content, and initial codes were assigned to each quote derived from posts and comments, which were clustered into six main themes: patients’ experiences with the syndrome, symptoms, searching for a doctor and relationship with healthcare professionals, pharmacological management, self-management, and impact of fibromyalgia.

## 3. Results

### 3.1. Participants

Females published the majority of posts on the page (95%). Although some posts were shared by anonymous authors, the feminine pronoun appeared in the context of the conversation, and the topic of discussion, such as pregnancy, reflected the gender of the participants. Some patients are highly educated and hold bachelor’s degrees or higher, as they share their educational background and work sector, such as working as physicians, pharmacists, nurses, and dieticians. However, the exact percentage is difficult to determine because educational status is not revealed for all participants.

### 3.2. Themes

Participants discussed various aspects of FMS and how it affected all aspects of their lives. These discussions revealed the following six themes (each with subthemes; Table 1): (1) patients’ experiences with the syndrome, (2) symptoms, (3) searching for a doctor and relationship with healthcare professionals, (4) pharmacological management, (5) self-management, and (6) the impact of fibromyalgia and peer support. Table 1 shows the themes and subthemes generated from the data, arranged according to the highest coverage based on the number and percentage of nodes.

#### 3.2.1. Patients’ Experience with Syndrome

##### Onset and Patients’ Own Explanation of the Syndrome

Patients often experience the onset of FMS following significant life events. For some women, “the illness started right after giving birth to the first child”, while another patient reported, “My pain started after three caesarean sections”. Another woman attributed her illness to miscarriage period “I was pregnant in the first year of marriage, and I miscarried, and then I started getting sick”. A male patient recognized the condition after chronic exposure to stress: “The problem started after being exposed to high work pressure for 2 years”. A significant traumatic life event was another reported cause: “Symptoms started when I lost my father in 2015, and they have continued to get worse”. A connection between the syndrome and coronavirus infection was noted in two patients. One of them stated, “I was diagnosed with fibromyalgia 6 months after being infected with coronavirus”. Most patients had primary fibromyalgia, with a few reporting autoimmunity. Many patients exhibited a good understanding of their illness and read scientific literature for updates. They explained the disease to each other in simple terms: “Fibromyalgia is considered a lifelong disease of the central nervous system” and “Patients with fibromyalgia have an amplifying response to pain”. They also understood that FMS is not an autoimmune disease; thus, no end-organ damage occurs. Moreover, patients discussed neurotransmitter-level abnormalities and their contribution to the symptoms.

##### Triggers of Symptoms

Most posts in the subtheme “triggers of the symptoms” indicate that stress is the leading cause of worsening symptoms. Many patients expressed their approach to avoiding stress by changing how they reacted to events and interacted with others. A female participant posted, “Now after I understood the nature of the disease, I stopped overreacting to the small stuff and started prioritising my health”. A confirming reply stated, “I learned to accept things I cannot change and control my reactions. I have also learned to put myself first.” Many patients believe that negative feelings, including anger, anxiety, and hyperempathy, were personal traits they had tried to change since the diagnosis because these negative feelings deteriorate any health improvement. One participant stated, “Anger brings us back to square one.” Another patient added, “Now, I dare to silence anyone who verbally hurts me and keep myself away from anxiety”. Moreover, some patients believe they suffer from hyperempathy toward others. “This illness makes a person very sensitive and empathise with everyone and everything. I have deep sadness over certain events such as the earthquake and strong empathy for the suffering of old people and children in particular”. This post received agreement from another patient: “I share with you the same feeling. I used to work in an oncology centre, and dealing with patients with cancer consumed me; as a result, I left my job”. Other triggers included hormonal fluctuations during the study period, severely cold or hot weather, and fasting during Ramadan. Many patients reported a worsening of their symptoms during Ramadan, when they had to fast for approximately 12–15 h in Arab countries. A female patient asked, “Are there people like me who cannot fast? I have been unable to fast for years and pay atonement.” One participant answered that she cannot fast: “Every year, I try for the first few days, but then, at the end of the month, I cannot get up from bed because of fatigue”. Another woman described her tiredness during Ramadan: “From the middle of Ramadan, my energy started to run out, and by the time of Eid, I started having a relapse that has not gone away until now”. A few participants expressed guilt for not attempting to fast during Ramadan, but others answered back with a phrase from the Qur’an: “Allah does not burden a soul beyond that it can bear”.

##### Severity and Treatability of Disease

Although most patients described themselves as tired and in pain, the severity of fibromyalgia differed among the patients who participated in the discussion. Some patients could grade their pain and fatigue intensity and link it to the type of activity they could accomplish or the time they spent on a task: “Walking needs a miracle”, “climbing stairs at home is painful”, “I can’t walk more than 10 min”. The more daily activity limitations a patient has, the worse they feel about themselves and their potential for recovery: “I am trapped in my body”. Interestingly, it is not uncommon for patients to describe the severity of the condition as if they were fighting a human being who lacks morality: “It is a fierce and immoral disease”. In contrast, patients who witness an improvement in their condition because of adherence to treatment or long-term self-management can similarly rate their quality of life: “I am 80% normal now! I no longer get tired easily, my sleep and memory have improved, and 80% of the pain has gone; I am free of medication and functional. However, I still have bad days from time to time”. Many patients exist between despair and hope but realize that the syndrome is incurable and acknowledge that a better quality of life can be achieved.

#### 3.2.2. Symptoms

**Figure 1:**
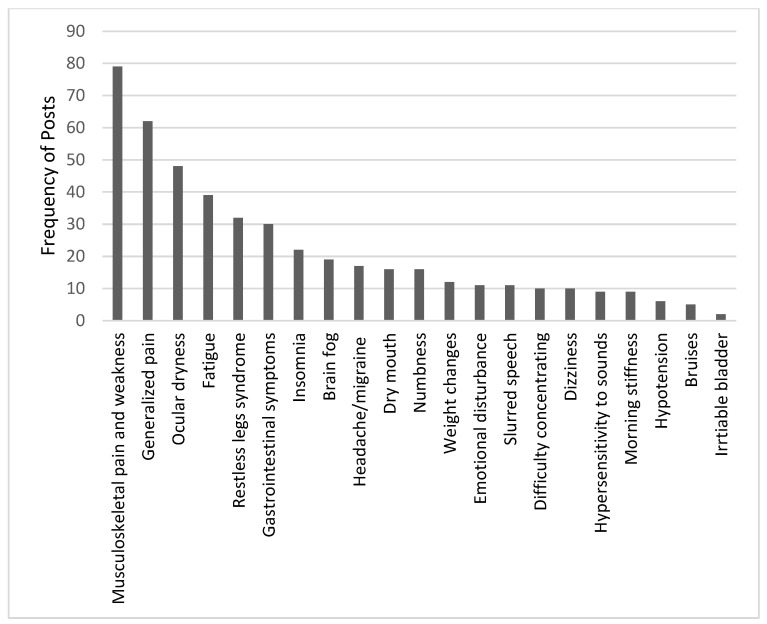
The frequency of symptoms discussed by patients with fibromyalgia.

Participants shared their experiences with symptoms before and after diagnosis. Posts about symptoms also included discussions by newly diagnosed patients seeking support from others with similar complaints, as well as those who suspected they might have fibromyalgia secondary to an autoimmune disease. The most common symptoms were musculoskeletal pain, weakness, generalized pain, and fatigue. Figure 1 shows the number of posts and discussions on symptoms. Symptoms affect all aspects of life, including personal, family, and career areas, and impair the ability to focus on duties and tasks. A female participant described how symptoms were affecting her: “Unfortunately, as I get older, I’m noticing that the symptoms are getting more intense, and the flare-ups are more frequent, last longer, and truly take a toll on me; being stuck in bed for days, my work is being affected”. The intensity of symptoms has been reported to worsen after coronavirus infection or vaccination, as in the following statement: “I was diagnosed with fibromyalgia a short time ago. I have complained of sleep disturbances. When I wake up in the morning, I feel stiff and tired. At work, I have brain fog. My symptoms worsened after I was infected with coronavirus”. Patients believe that some symptoms are an integral part of the syndrome and that no patient escapes them. Of these, irritable bowel syndrome (IBS) is the most common. A female patient posted asking if there is a relationship between fibromyalgia and IBS, and several comments agreed and considered the relationship a fact: “If someone does not have irritable bowel syndrome, then he does not have fibromyalgia”. Another continued: “A relationship? They are close friends”.

#### 3.2.3. Pharmacological Management of Fibromyalgia

Several classes of medications, including painkillers, antidepressants, anticonvulsants, and muscle relaxants, are prescribed to patients with fibromyalgia. The frequency of drug posts, their classification, and possible benefits for fibromyalgia are shown in Table 2. Duloxetine, gabapentin, pregabalin, and amitriptyline were the most frequently discussed medications among patients. Posts discuss the efficacy of one drug in comparison with other classes, dose, duration of treatment, and side effects, and share successful stories of therapeutic management. Most patient discussions revealed that they did not adhere to the prescribed treatment protocol. Barriers to drug adherence include the fear of addiction, concerns that antidepressants may increase suicidal thoughts, and intolerance to side effects. One patient stated, “I took amitriptyline for more than a year, then I withdrew it and stopped all other medications because it always made me sleepy and lethargic, and I felt disconnected from the world. The problem with these medications is that they are addictive”.

Among the overwhelming side effects that cause drug rejection, weight gain was reported by many patients and imposed physical and mental burdens: “I have been taking medications for fibromyalgia for 20 years. I gained a lot of weight from the medications and suffered from many side effects. I only woke up a few months ago to realise that everything I was suffering from was because of the drugs. I have stopped taking them now, and my weight is back to normal, and the stomach pain and many symptoms have disappeared.” Fear of end-organ damage was another reason for stopping the medication. One patient advised: “Stop all analgesics; they cause renal failure”. Moreover, participants often stopped medication early in the treatment because they thought the drug lacked efficacy, believing that medications lose their efficacy after a while: “I took duloxetine for a month, and it successfully controlled my pain, then stopped working”. A few patients also declared that they feel the stigma of using drugs originally prescribed as anticonvulsants or antidepressants because the notion that fibromyalgia is of psychological origin brings negative feelings to many women. Those who stopped medication were switched to over-the-counter analgesics such as paracetamol and ibuprofen.

#### 3.2.4. Searching for a Doctor and Relationship with Healthcare Professionals

Fibromyalgia was diagnosed by neurologists or rheumatologists, as revealed by the majority of participants. Many posts in this theme (44%) requested referrals to an appropriate physician based on other patients’ experiences. Numerous names and addresses were shared across different regions and countries, with details about the physician’s expertise, treatment methods, personality, and approach to dealing with patients. Both negative and positive comments were openly shared to benefit others. A physician is complimented when they possess attributes such as being an excellent listener to the patient’s complaints, discussing the patient’s concerns, understanding fibromyalgia symptoms, and having a treatment strategy tailored to the patient’s needs.

However, many patients criticized physicians who do not believe that fibromyalgia is a real disease originating from physiological abnormalities rather than a mental disorder. One patient stated, “An illiterate doctor does not recognise fibromyalgia as a real disease and classifies the syndrome as a psychological illness. In addition to our pain, the lack of understanding and recognition of the disease leads to depression”. Some patients decided not to seek medical care because they believed that “doctors have nothing but antidepressants, tranquilisers, anti-epileptic drugs, and painkillers. They give similar advice, and each doctor objects to and contradicts other doctors’ treatments”. Others think that doctors “care about money” and are “testing different drugs on patients”. Moreover, patients complained that physicians did not educate them about the disease: “he prescribed a drug and did not talk to me about fibromyalgia”.

#### 3.2.5. Self-Management and Lifestyle Modification

**Figure 2:**
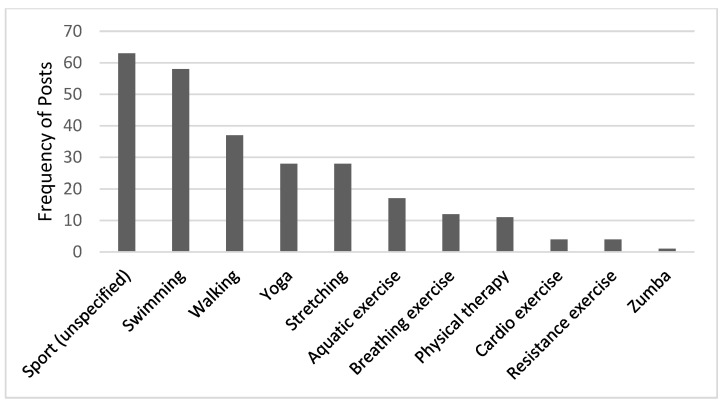
Type of physical activities reported by patients with fibromyalgia.

Patients with fibromyalgia have reported various lifestyle modifications to manage disease symptoms, especially when they choose to stop medication and avoid doctor visits. Many patients report that altering long-term habits, primarily diet and physical activity, and maintaining these modifications for months or even years has improved their physical and mental health. Dietary modifications that include taking over-the-counter supplements, eliminating dietary constituents such as gluten, and using herbs are shown in Table 3. Many patients believe they suffer from food intolerances and eliminate certain dietary components or follow specific dietary plans. This decision is based on a self-monitoring of foods that cause reactions or FoodPrint testing for patients who can afford it. Most patients limit or eliminate three main components: gluten, sugar, and dairy products. Posts discussing removing gluten received the highest frequency (*n* = 118), as many patients believe that removing gluten relieves IBS symptoms. The frequency of posts about magnesium as an over-the-counter dietary supplement was the highest compared to other supplements (*n* = 103). It has gained much interest among patients to relieve pain from muscle spasms, brain fog, anxiety, and to aid sleep. The choice of physical activity depends on each patient’s access to specific sports, physical ability, and pain tolerance. In several posts, patients discussed including sport in lifestyle modification without mentioning the type (*n* = 63). Others have specified swimming (*n* = 58), walking (*n* = 37), yoga (*n* = 28), stretching (*n* = 28), aquatic exercise (*n* = 17), and breathing exercises (*n* = 12). Moreover, physical therapy, resistance exercise, and Zumba were discussed to a lesser extent (Figure 2). Traditional practices of cupping therapy (*n* = 16) and acupuncture (*n* = 2) were also recommended by a few patients. Several posts convey recommendations for combining diet, supplements, and exercise to achieve a balanced life and long-term control of fibromyalgia symptoms. “Intermittent fasting, a healthy diet, gluten-free, dairy-free, sugar-free, exercise—especially yoga, swimming, and walking—and special supplements like magnesium, vitamin D, and omega-3 make a big difference”.

#### 3.2.6. Impact of Fibromyalgia and Peer Support

Fibromyalgia affects mental health, with participants describing and sharing emotional pain, worries, and concerns about themselves, their families, and their careers. Most participants experienced negative emotions and felt isolated from family, friends, and the community, with a lack of understanding and support from those around them being the main reason.

Family planning and the decision to become pregnant were affected by the syndrome. Some women shared fears of exacerbating symptoms during pregnancy while stopping medication: “I have been delaying pregnancy for a couple of years now out of fear. I am getting older and want to become a mother”. Chronic pain and stiffness caused anxiety about hypothetical scenarios, as experienced by one female participant: “I have an idea that dominates my mind whenever I try to cross the street. I fear that my muscles will betray me at the wrong moment. Although this has never happened, I cannot control my fear”.

Participants described how the syndrome impaired their ability to attend work that required long hours or physical activity, such as standing for long periods. This led to late arrivals at work, career changes, or resignations. One male patient shared his difficulties at work: “I got a warning at work because I was late. They don’t understand that I am sick; they think I am faking my illness. Even my family keeps telling me that I am not ill. Unfortunately, everyone around me doesn’t support me. If I get two more warnings, I’ll be fired. This will make my financial situation difficult, and the treatments are expensive”. Peer support was evident in the group, with participants advising each other on how to overcome difficulties and offering compliments and supportive messages that conveyed an understanding of others’ suffering. In many instances, religious sayings, prayers, and words of encouragement and comfort are posted in response to someone’s pain or sadness. Stigma can be recognized from the posts under this theme where perceived stigma that reflects patients’ belief that family members and people from their surroundings have negative thoughts about them received the highest frequency. Self-stigma is also present in some patients’ statements, where they have low self-worth and low self-esteem, negative attitudes, and label themselves with words like: “ I am no longer effective” or “I am like Alzheimer’s patients”, referring to symptoms like forgetting, brain fog, and low concentration. Stigma from healthcare professionals, that leads to insufficient care and occupational stigma, were also reported by a few patients (Table 4).

## 4. Discussion

This study explored the perceptions of patients with fibromyalgia from Arab countries regarding their chronic illness experience through a content analysis of data from an open-access Facebook support group. The study findings reveal the patients’ journey from seeking a diagnosis and searching for a doctor until stopping treatment, as reported by many, and following a personal holistic approach to managing the syndrome. Most patients were women, as the syndrome is reported to have a high female predominance (approximately 80%) [15]. Patients reported having primary fibromyalgia, while a few patients revealed the presence of associated disorders such as rheumatoid arthritis, Sjogren’s syndrome, ankylosing spondylitis, and multiple sclerosis. In some cases, patients were treated for fibromyalgia before the discovery of autoimmunity, and vice versa. This overlap has been reported in previous studies due to symptom similarity and the absence of clinical laboratory tests confirming the presence of fibromyalgia [37]. Moreover, a few patients reported the onset of FMS after pregnancy and cesarean section. This is consistent with a previous study by Saa’d et al., who reported the prevalence of FMS symptoms among 27% of full-term healthy pregnant women who fulfilled the ACR criteria in the study and that affected the course of delivery and the need for anesthesia, despite not being diagnosed with FMS before the recruitment [38]. In a retrospective cohort study comparing the outcomes of pregnancies of women with and without FMS, the authors reported that patients had higher rates of intrauterine growth restriction [39]. Neuro-hormonal fluctuation during the menstrual cycle and pregnancy, especially in the third trimester and postpartum, was hypothesized to cause CNS sensitization, in which the inhibitor gamma-aminobutyric acid (GABA) neurotransmitter is reduced with sex hormone level fluctuation. At the same time, other excitatory neurotransmitters may rise [40]. FMS may develop as a sequela of different stressors, such as coronavirus infection, which causes severe inflammatory responses referred to as “cytokine storms”, and this is thought to sensitize the central and peripheral nervous system [41]. The journey to diagnosis is painful, as data show that many patients reported spending 5–11 years visiting physicians of different specialties. Delayed diagnosis and its negative consequences have been documented previously [11,12]. Receiving a diagnosis brings temporary relief, as almost every patient undergoes drug rotation. Finding a drug that relieves multiple symptoms persisting for years is challenging. This is consistent with other qualitative studies on patients with fibromyalgia that have reported the psychological impact of delayed diagnosis [42,43,44].

The participants described a wide range of debilitating physical symptoms that varied in duration and intensity. Chronic widespread pain, including musculoskeletal pain, received the highest ratings from patients. Patients believe in the interconnection between IBS and FMS, and many reported living with IBS and food intolerance, which required lifestyle modifications such as the elimination of gluten and dairy products. Interestingly, IBS and FMS are both prevalent in women and share similar predisposing factors such as emotional trauma, depression, and anxiety [45]. The proposed pathogenic mechanism is an improper transmission along the gut–brain axis owing to the disturbance of gut microbiota, which affects the metabolism of GABA and may alter the GABA/glutamate balance seen in FMS [46]. Patients reported that stress is the primary triggering factor that precipitates attacks and intensifies pain. Many conveyed that avoiding negative feelings associated with situations and people, even relatives, has become a life strategy for managing the syndrome. Many people described themselves as sensitive and reactive to their surroundings, and any traumatic event imposed a mental and physical burden that manifested as pain and fatigue. In many patients, the onset of fibromyalgia is linked to tragic life events. Consistent with our findings, other studies have suggested an association between psychological trauma and fibromyalgia [42,43]. Being emotional and overly empathetic is a personal trait that could be a vulnerability factor to personal distress. Studies in cognitive neuroscience have uncovered the brain circuits that become active when individuals respond to pain in an empathetic context [47]. An experimental study by De Tommaso et al. recorded the emphatic nociceptive paradigm of patients with FMS using the electroencephalogram compared to healthy subjects. Findings indicate that when patients with FMS empathize with someone else’s pain, the activation of the somatosensory circuit is triggered, closely resembling experiencing their own pain [48].

In our study, participants discussed the overwhelming experience of finding a physician who listens to and supports the assessment and treatment processes and shared opinions about how healthcare professionals lack knowledge and experience of the syndrome. Patients feel unsupported when physicians deny fibromyalgia as a valid diagnosis and attribute the symptoms to psychological factors. This adds to their social isolation and loneliness, as they receive similar dismissive perspectives from family members, friends, and co-workers. This leads to stigma, with patients being labelled as delusional and accused of complaining despite being healthy. This, presumably, explains the noncompliance of patients to antidepressant prescriptions, in addition to the side effects. In agreement with our findings, previous qualitative studies have reported patient frustration with the healthcare system due to the failure to recognize and diagnose fibromyalgia, the lack of experience in managing the condition, and the insufficient patient education provided by physicians [42,43,44,45,46,47,48,49].

In our study, fluoxetine, pregabalin, gabapentin, and amitriptyline were the most frequently discussed medications. Patients withdrew from treatment without consultation due to fears of addiction and side effects. Others believed that these drugs were ineffective and no longer reduced pain. Some patients reported that reading the inserted pamphlet on antidepressants and anticonvulsants led to noncompliance because of the indications and adverse reactions. This is consistent with Aster et al. who conducted a cross-sectional study and found that NSAIDs, amitriptyline, and pregabalin were among the current and past medications used by patients with fibromyalgia [15]. The causes of discontinuation reported in that study align with our findings [15]. In accordance with previous research [50], patients in the current study developed coping strategies to manage the syndrome, such as diet, physical activity, and over-the-counter pharmaceutical preparations, while reducing or discontinuing prescribed medication. Most patients adopted a balanced approach, gradually combining lifestyle modifications, especially when introducing a variety of sports and physical activities to avoid pain and exertion. Similar coping strategies have been reported previously.

Sharing experiences within a supportive group has positive effects, with some patients describing it as finding a “big family” or a “tribe”. Older or more experienced participants, especially those with healthcare or medical backgrounds, served as mentors who provided comments and advice to newer members of the group. Patients felt that their voices were heard, and they could freely ask questions, explore all aspects of their illness, and find explanations for how they felt physically and mentally. This finding agrees with that of Reig-Garcia et al. who explored the impact of a peer social support network on women with fibromyalgia [51]. The authors reported that peer support had multiple positive effects on physical, mental, and social well-being.

One of the main limitations of this study was that the data collection from Facebook does not provide any information about participants’ demographic data. Moreover, there is a lack of information regarding the participants’ medical history, such as the disease duration, diagnosis criteria, medications, and co-morbidities. The data presented in this study were dependent on what participants revealed about themselves in the posts, making it impossible to ensure that all subjects would meet the ARC criteria for diagnosis and making it possible for the supportive group to have members with somatic symptom disorder who were mistakenly diagnosed with FMS. Another limitation is that a single researcher performed data collection and analysis. However, Bradley et al. indicated that experts in the field argued that a single researcher who conducts the coding process is sufficient and preferred [52,53,54,55]. Unlike interviews, the text analysis process did not involve data transcription; therefore, a peer review was unnecessary to ensure credibility and reduce bias [52]. A single researcher who collects and analyzes data is performing an integrated process that is essential for obtaining high-quality data, especially in research where maintaining ongoing relationships with study participants is necessary [52]. Moreover, the current content analysis is more descriptive than interpretive, and the posts do not have multiple meanings; thus, the codes and themes are easily identified, and the author expects a high agreement between coders if another coder is involved. One approach utilized in this study is that the author started the research by observing the content and returning to data at multiple time points to ensure the consistency of data coding. The second approach was a peer debriefing, in which one expert colleague was involved for just one session during data analysis to discuss the methods and interpretation of data.

## 5. Conclusions

In conclusion, patients with fibromyalgia in Arab countries share similar health journeys and experiences with patients from other cultures. In addition to the overwhelming physical symptoms, the syndrome has psychological, social, and economic impacts that affect quality of life. Many patients oppose the therapeutic management of fibromyalgia and have developed coping strategies to avoid the adverse effects of medication, the cost of therapy, and the unsupportive healthcare system. Socio-cultural differences in family responsibilities impose specific duties on Arab women, such as caring for the children as a daily sole carer and other responsibilities toward the spouses’ family. These factors were triggering stress for some women in our study. Moreover, there is also a religious duty, such as during the month of Ramadan, which many reported as a cause of health deterioration, struggle, and enduring guilt for not being able to fast. Practitioners involved in patient care should possess the necessary clinical training and skills to support patients’ needs from diagnosis to treatment. In some patients, the pathology is linked to traumatic events and life experiences; thus, FMS sufferers may benefit from counselling, cognitive behavioral therapy, or psychotherapy to address personality traits or psychological trauma. Moreover, community health programs and awareness activities are needed to support patients and would be beneficial in terminating the social isolation of patients with the syndrome.

## Figures and Tables

**Table 1:** Themes and subthemes of posts and comments.

Theme	Explanation	Subthemes	Number of Nodes (%)	Number of Words
Self-management	Posts discussing self-treatment with over-the-counter medications and lifestyle modifications, including diet and exercise.	Diet, exercise, vitamins, herbs, over-the-counter medications.	772 (28.51)	16,159
Symptoms	Posts discussing current symptoms shared by patients.	Pain, fatigue, musculoskeletal pain and weakness, gastrointestinal issues, ocular issues, weight changes, insomnia, memory, and mood issues.	448 (16.54)	7885
Impact of fibromyalgia and peer support	Posts discussing the emotional impact of the syndrome, stigma, and how patients support each other.	Feeling down, feeling isolated, feeling guilty, concern about disability, impact on family, others’ misunderstanding, need for support, stigma, patient-to-patient support, seeking advice from others.	443 (16.40)	10,927
Pharmacological management	Posts discussing the efficacy of drugs, both positive and negative, and compliance.	Efficacy, sharing experiences with a brand, dose, duration of treatment, recommendations, compliance, starting and stopping medications.	432 (15.95)	8320
Patients’ experiences with the syndrome	Posts discussing patients’ experiences with the syndrome.	Patients’ own explanations of the syndrome, onset of the disease, triggers of symptoms, severity of disease, beliefs in treatability, differential diagnosis.	333 (12.30)	7555
Searching for a doctor and relationship with healthcare professionals	Post used to seek advice about finding the right healthcare professional.	Asking for help to find a doctor, positive and negative opinions about treating physicians.	279 (10.30)	4881

**Table 2:** Number of posts about therapeutic management of fibromyalgia.

Drug	Class	Uses and Possible Benefits in Fibromyalgia	Frequency of Posts (*n*)	Example of Patients’ Statements
Duloxetine	Selective serotonin and norepinephrine reuptake inhibitor	Approved for chronic musculoskeletal pain, anti-anxiety, fibromyalgia [24]	140	“I am on Duloxetine for two years now, side effects disappeared after two weeks of use. I will continue using it.”, “I suffered from most of Duloxetine side effects including constipation”. “I have a child with special needs, and I have to take care of him, even if it means taking duloxetine for life to overcome physical pain and exertion.”
Escitalopram	Selective serotonin reuptake inhibitor	Major depressive disorder	7	“I have been on escitalopram for two years now. I have no side effects”.
Vortioxetine	Serotonin modulator	Major depressive disorder	1	“Vortioxetine is a great drug and has no mentioned side effects.”
Amitriptyline	Tricyclic antidepressant, serotonin, and norepinephrine reuptake inhibitor	Major depressive disorder, off-label use for fibromyalgia and chronic pain, irritable bowel syndrome, migraine [25]	30	“Amitriptyline causes mouth dryness, which was bothersome, and I stopped the drug”, “Amitriptyline made me sleepy, fatigued, and nervous. I stopped the drug after the second dose”
Quetiapine	Atypical antipsychotic; it blocks dopamine D2 and serotonin 2A (5HT2A) receptors	Approved for schizophrenia, off-label use for insomnia and anxiety [26]	1	“Without quetiapine, I would lose my mind because of lack of sleep”.
Amitriptyline hydrochloride/Perphenazine.	First-generation antipsychotic: blocks dopamine D2 receptors/serotonin and norepinephrine reuptake inhibitor	Tranquilizer and antidepressant, off-label use for fibromyalgia to manage pain and sleep problems [27]	1	“After a series of trial with anti-depressants, the doctor prescribed Amitriptyline/Perphenazine to control my pain”
Flupentixol/melitracen	Antipsychotic: blocks dopamine D1& D2 receptors/serotonin and norepinephrine reuptake inhibitor	Tranquilizer and antidepressant, off-label use for fibromyalgia to manage pain and sleep problems [27]	2	“I can tolerate Flupentixol/melitracen but not duloxetine or amitryptyline”.
Venlafaxine	Serotonin and noradrenaline reuptake inhibitor	Major depressive disorder, social anxiety disorder, panic disorder, off-label use for fibromyalgia and migraine prevention [28]	1	“I took pregabalin for two years and tapered it. I was on duloxetine for a while. Now, the doctor has given me venlafaxine.”
Gabapentin	Anticonvulsant	Neuropathic pain, restless legs syndrome, off-label use for fibromyalgia and mood disorders [29]	52	“The improvement was almost temporary in the first month. The pain gradually returned. Then, the doctor increased the dose of the medication and I continued on this rollercoaster for 8 months, and finally I decided to stop it”. “Gabapentin causes addiction”, “Gabapentin made my symptoms worse”, “Gabapentin causes insomnia and made me nervous”, “I am always hungry”
Pregabalin	Anticonvulsant	Neuropathic pain, seizures, fibromyalgia, off-label use for restless legs syndrome, insomnia, anxiety disorders [30]	55	“pregabalin makes me hallucinating”, “pregabalin causes addiction on long-term use”, “pregabalin increased my weight”
Carbamazepine	Anticonvulsant	Used for epilepsy, off-label use for fibromyalgia, neuropathic pain, and restless leg syndrome [31]	2	“I take carbamazepine is given to relieve spasm, migraine and to sleep.”
Bisoprolol	Beta1-blocker	Hypertension and heart disorders, non-FDA approval for migraine, arrhythmia, and anxiolytic use [32]	4	“Bisoprolol did not work well with me. I take another beta-blocker which control my tachycardia but causes hypotension”
Nebivolol			1	“I took nebivolol, which is similar to bisoprolol. It has controlled the heart rate temporarily, but palpitations remained; I continued using it for a few months, and then I stopped it on my own without consultation”.
Diazepam	Anxiolytic benzodiazepine	Management of anxiety disorders: off-label for muscle spasms, pain, and insomnia [33]	1	“Before diazepam, I could not sleep for days”
Lorazepam			1	“A neurologist prescribed lorazepam for me. I started with half a tablet. I have taken one tablet for the last 5 years before bedtime. If I skip a dose, I can’t sleep because of the pain”
Bromazepam	Short-acting benzodiazepine	Approved for anxiety	1	“Yesterday, my doctor prescribed bromazepam and duloxetine to help me relax and sleep”
Zolpidem	Non-benzodiazepine receptor modulator	Approved for insomnia	1	“I took Zolpidem to sleep. It helped me for a while, then stopped working”
Lithium	Mood stabilizer	Approved for mania, off-label use for pain and insomnia [34]	1	“Lithium controls my panic attack”
Clonidine	Alpha-2- adrenergic agonist	Approved for hypertension, off-label use for anxiety and insomnia [35]	1	“I recently started taking clonidine. It is used to manage blood pressure, but now scientists have discovered that it is effective for anxiety. I have been taking it for a few days, and the pain has completely disappeared. I am surprised!”.
Muscle relaxants				
Muscle relaxants (unspecified class)			22	
Cyclobenzaprine	Centrally acting skeletal muscle relaxant	Approved to relieve muscle spasms, off-label use for fibromyalgia to manage insomnia [36]	1	“When I am in a lot of pain, I take Cyclobenzaprine at night”
Tolperisone		Approved to relieve muscle spasms	1	“I tried tolperisone, but muscle pain did not subside”.
Paracetamol/orphenadrine	Non-opioid analgesic/muscle relaxant	Approved to relieve muscle spasms	1	“ I believe that paracetamol/orphenadrine is the right muscle relxant for fibromyalgia”
Analgesics				
Tramadol	Opioid analgesic	Analgesic	4	“I take tramadol once weekly and any analgesic containing codeine. Other analgesics did not control my pain”.
Short-term cortisone	Corticosteroid	Analgesic/Anti-inflammatory	4	“I take cortisone for a short period until the attack subsides. 30 tablets will not cause significant complications but will relieve the pain.”
Ibuprofen	Nonsteroidal anti-inflammatory drugs (NSAIDs)	Analgesic/Anti-inflammatory	30	“I have been suffering from unexplained bruises, but they were gone when I stopped taking ibuprofen and other NSAIDs. They interact with my treatment”.
Diclofenac			5	“ I take diclofenac to relieve the pain. It is very effective”, “I need a muscle relaxant and diclofenac to control flares”
Nimesulide			2	“I take nimesulide intermittently “
Celecoxib			4	“I take celecoxib and muscle relaxant. Gabapentin did not help me”.
Etoricoxib			3	“Etoricoxib controls my pain”
Paracetamol	Non-opioid analgesic	Analgesic and antipyretic	50	“I only take Panadol Joint when the pain is unbearable”, “Paracetamol is the best with no side-effects”.
Paracetamol/Ibuprofen/Caffeine	Non-opioid analgesic/NSAIDs	Analgesic	10	“I take Paracetamol/Ibuprofen/Caffeine intermittently for muscle pain”
Ibuprofen/Paracetamol	NSAIDs/Non-opioid analgesic	Analgesic	10	“I stopped all medications years ago, but I recommend ibuprofen/paracetamol when necessary.”

**Table 3:** Dietary modification approaches of patients with fibromyalgia.

	Type	Frequency of Posts (*n*)
Supplements: vitamins and minerals	Multivitamins	10
	Magnesium (malate, bisglycinate, L-threonate)	103
	Vitamin D	56
	B-complex	30
	B12	28
	5HTTP	28
	Iron	20
	Melatonin	20
	Omega	14
	Zinc	10
	Q10	6
	Vit C	6
Elimination of dietary constituents	Gluten	118
	Sugar	81
	Milk	44
Diet	Keto	5
	Low carb	5
	Vegan	5
	Intermittent fasting	11
Herbs	Ashwagandha	13
	Moringa	4
	Costus	4
	Wheatgrass supplement	4

**Table 4:** Types of perceived stigma by fibromyalgia patients.

Type of Stigma	Description	Frequency of Posts (*n*)	Example of Patients’ Statements
Perceived stigma	Patients’ believe that others have negative beliefs about them, like considering them delusional and mentally ill.	60	Patient 1: “Our problem is not only with the doctors. The problem is that society must accept us and improve our living requirements” Patient 2: “People around me thought I was faking and lying about my symptoms and described me as a lazy person.”
Self-stigma	Patients perceive themselves as weak, incompetent, and a burden, and have lower self-esteem and negative belief about self.	38	Patient 1: “I feel weak. I have to bury all my dreams because of this disease.” Patient 2: “I have thoughts that I am not a good mother, and I think obsessively about how my husband’s family think about me.”
Healthcare professional stigma	Healthcare professional under-treat patients with fibromyalgia and think about their condition as a mental disorder.	17	Patient 1: “I had an accident, and I was hospitalized and suffered pain for which I asked the physician for severe painkillers; the physician refused to believe the severity of my pain as a fibromyalgia patient and treated me as an addict to painkillers, threatening to stop the treatment.” Patient 2:“My doctor does not believe in fibromyalgia, and he told me it is a lie, and women believed it. If you are sick, then the lab should confirm that”
Occupational stigma	Patients are treated in the workplace as incompetent or mentally-ill due to low productivity	6	“I am a schoolteacher and love my job, but I was treated as someone who could not bear the work burden. I feel pressured to get early retirement, but staying at home between four walls will worsen my situation.”

## Data Availability

The original contributions presented in this study are included in the article. Further inquiries can be directed to the author.
